# Experiences of women who travel for abortion: A mixed methods systematic review

**DOI:** 10.1371/journal.pone.0209991

**Published:** 2019-04-09

**Authors:** Jill Barr-Walker, Ruvani T. Jayaweera, Ana Maria Ramirez, Caitlin Gerdts

**Affiliations:** 1 ZSFG Library, University of California, San Francisco, San Francisco, California, United States of America; 2 Ibis Reproductive Health, Oakland, California, United States of America; 3 Division of Epidemiology, School of Public Health, University of California, Berkeley, Berkeley, California, United States of America; USC Keck School of Medicine, Institute for Global Health, UNITED STATES

## Abstract

**Objective:**

To systematically review the literature on women’s experiences traveling for abortion and assess how this concept has been explored and operationalized, with a focus on travel distance, cost, delays, and other barriers to receiving services.

**Background:**

Increasing limitations on abortion providers and access to care have increased the necessity of travel for abortion services around the world. No systematic examination of women’s experiences traveling for abortion has been conducted; this mixed-methods review provides a summary of the qualitative and quantitative literature on this topic.

**Methods:**

A systematic search was conducted using PubMed, Embase, Web of Science, Popline, and Google Scholar in July 2016 and updated in March 2017 (PROSPERO registration # CRD42016046007). We included original research studies that described women’s experiences traveling for abortion. Two reviewers independently performed article screening, data extraction and determination of final inclusion for analysis. Critical appraisal was conducted using CASP, STROBE, and MMAT checklists.

**Results:**

We included 59 publications: 46 quantitative studies, 12 qualitative studies, and 1 mixed-methods study. Most studies were published in the last five years, relied on data from the US, and discussed travel as a secondary outcome of interest. In quantitative studies, travel was primarily conceptualized and measured as road or straight-line distance to abortion provider, though some studies also incorporated measures of burdens related to travel, such as financial cost, childcare needs, and unwanted disclosure of their abortion status to others. Qualitative studies explored regional disparities in access to abortion care, with a focus on the burdens related to travel, the impact of travel on abortion method choice, and women’s reasons for travel. Studies generally were of high quality, though many studies lacked information on participant recruitment or consideration of potential biases.

**Conclusions:**

Standardized measurements of travel, including burdens associated with travel and more nuanced considerations of travel costs, should be implemented in order to facilitate comparison across studies. More research is needed to explore and accurately capture different dimensions of the burden of travel for abortion services on women’s lives.

## Introduction

The World Health Organization estimates that one in four pregnancies ends in abortion [1], yet access to this essential service is limited globally. Abortion is legally permitted only in cases where a woman’s life is in danger or prohibited altogether in 66 countries; however, even in the 56 countries where abortion is technically available on request, restrictive abortion laws can make accessing abortion difficult [2–4]. Conscientious objection, the refusal to participate in abortion services because of religious, moral, philosophical or ethical beliefs, also affects access to abortion. Conscientious objection is legal in 21 European countries [5], including Italy, where 70 percent of gynecologists are conscientious objectors [3], and is practiced in the United States where it is federally protected under the “Church Amendments”[6]. In the United States, 90 percent of counties do not have access to an abortion provider [7]; such limited accessibility often necessitates travel for abortion services.

Traveling for abortion is not a new phenomenon; Irish and Canadian women have been traveling to the United Kingdom and the United States, respectively, to access abortion services since the 1960s [8]. More recently, restrictive measures including Targeted Regulation of Abortion Providers, or TRAP laws, have limited abortion access in many parts of the United States; one half of states have experienced a decline in the number of abortion facilities in the past 5 years, with some regions experiencing decreases up to 18 percent [7]. A recent study identified 27 major US cities that lacked a publicly advertised abortion facility within 100 miles, indicating that women across the United States must travel long distances for abortion services [9]. Even within states, there are large variations in abortion provider access, indicating spatial disparities that reflect a lack of access for women in rural areas [10]. With such limited provider options for women close to home, travel is often a necessary step in obtaining abortion services, but it does not come without costs. Studies from several states have reported negative effects related to abortion travel, including increases in travel time, transportation and childcare costs, stigma resulting from the need to disclose the abortion to others, and delays in care [11–15].

Given increasing limitations on abortion access in the United States, Europe and elsewhere [2, 16], studying women’s experiences of traveling for abortion is more important than ever. Despite an increase in research on this topic in recent years, there has been no comprehensive analysis of travel for abortion services. This paper aims to systematically examine the breadth and depth of the published literature on women’s experiences traveling for abortion services by assessing different methodological approaches used and highlighting the importance of looking at travel in this field. While we cannot establish an overall description of travel and abortion due to the inconsistencies in methodologies and outcomes of the reviewed studies, we aim to assess how this concept has been explored and operationalized in order to show the need for consistent measurement of travel outcomes when accessing abortion and to provide recommendations for researchers on how to assess travel for abortion in future studies.

## Methods

### Search strategy

A PROSPERO protocol was registered for the review (#CRD42016046007) [17] and PRISMA guidelines were followed [18]. A search strategy was created by the first author, a clinical librarian (JBW), using keywords and controlled vocabulary, including MeSH, for the concepts of abortion and travel. The systematic search for articles on travel for abortion services was conducted on July 18, 2016 in PubMed, Embase, Web of Science, Popline, and Google Scholar. As this is a rapidly growing area of research, we re-ran our search on March 20, 2017 to ensure that new publications were included in the review. No date or language limits were used and unpublished and grey literature were not included. Detailed search strategies can be found in Appendix 1.

### Study selection

Studies were excluded if they were not in English, did not contain original analysis, did not present data around travel for abortion, or involved a participant group other than women seeking or obtaining abortion services. Articles were double screened by two reviewers (JBW & RJ) based on title and abstract to determine if they met the inclusion criteria for full-text review. Articles without abstracts that appeared potentially relevant based on title were moved to final screening for further consideration. Full text screening was completed by two reviewers (JBW & RJ); each author screened all articles, and discrepancies were resolved by a third reviewer (CG).

### Data extraction

Standardized forms were created to extract relevant data from the studies, including study setting, population, methodology, exposure and outcome measures, and results and conclusions related to abortion travel. Quantitative studies were categorized as *retrospective* if they relied on existing data sources such as hospital, clinic, or medical billing records; studies were considered to use *prospective* data collection if they enrolled abortion clients when they obtained or were seeking an abortion. We categorized studies as *longitudinal* if data was collected from participants at two or more time points; *cross-sectional* studies relied on only one data point per individual; *ecological* studies presented aggregate counts of the number of abortions or number of abortion clients at the facility, county, state, or national level. Studies were critically appraised using the STROBE checklist for quantitative studies, CASP checklist for qualitative studies, and MMAT checklist for mixed methods studies [19–21]. Data extraction was completed by research assistants and quality checked by two reviewers (JBW & RJ). Critical appraisal was completed independently by two reviewers (JBW & RJ) after completing consensus checks to ensure inter-rater reliability. Data extracted from quantitative and qualitative studies can be found in Appendices 2 and 3, and randomized critical appraisal data can be found in Appendices 4 and 5.

A thematic synthesis approach was used to analyze and synthesize the qualitative data. As our main research question was to understand how travel related to abortion was conceptualized and studied in the literature, we used an open coding process to develop salient themes. The results section from 12 qualitative studies and the qualitative results section from 1 mixed-methods study were uploaded into Dedoose, a web-based qualitative analysis software. Two authors (RJ & AR) conducted initial open coding on ten of the selected articles until saturation was reached, focusing only on results related to travel for abortion services. After substantial discussion, two authors (RJ & AR) grouped codes into second-order and first-order codes, with first-order codes representing overarching themes comprising both second-order and third-order codes (Appendix 6). These codes were applied to all qualitative findings. Any discrepancies between coding application were flagged and resolved through discussion. As all studies included in this review explicitly refer to study participants as female/woman, we use the words “female/woman” and the pronouns “she/her” throughout this paper. However, we acknowledge that some individuals who do not identify, as women are capable of pregnancy and may need timely access to safe abortion; while the perspectives of these individuals are not represented in the studies included in this review, findings may be relevant for these individuals as well.

## Results

### Study selection

**Fig 1:**
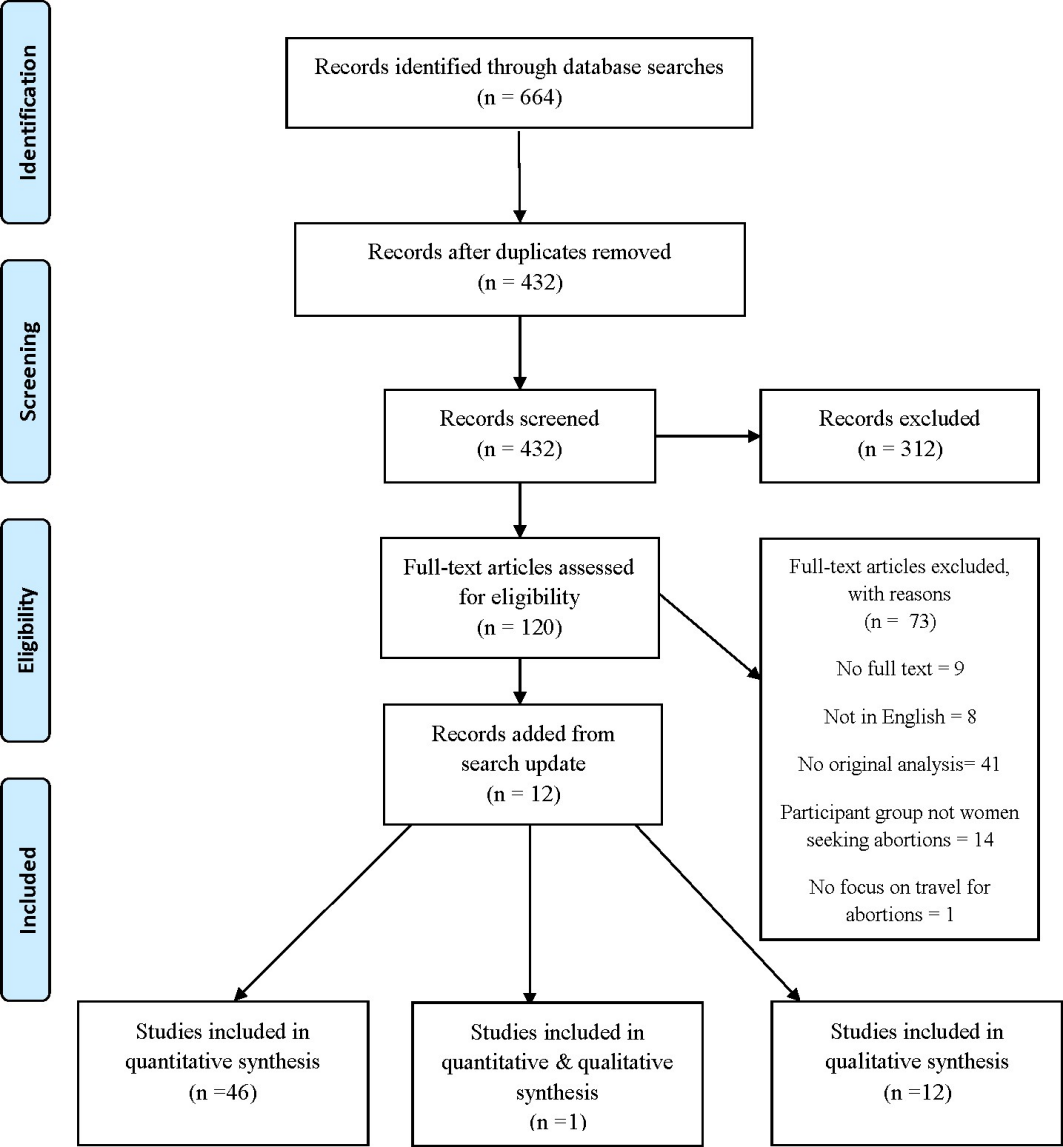
PRISMA chart. Study selection flow chart for final inclusion in analysis. [18].

The systematic literature search yielded 664 articles. After excluding duplicates, 432 articles were screened for inclusion based on title and abstract. The full text of 120 articles was assessed for eligibility, and 73 were eliminated based on established inclusion and exclusion criteria. The search update on March 20, 2017 yielded an additional 12 studies for review. Fifty-nine studies were included in the final analysis: 46 quantitative studies, 12 qualitative studies, and 1 mixed-methods study (Fig 1).

### Study characteristics

Table 1 describes the characteristics of the studies included in the review. The majority of studies (34 out of 59) used a cross-sectional design. Studies were published between 1975 and 2017, with the majority of studies (56%) published in the last five years, and a substantial number (18%) published more than 20 years ago. Over 90% of qualitative studies were published in the last two years. Studies represented findings from 11 countries, primarily from the United States (40 studies), Australia (five studies), the United Kingdom (five studies), and Canada (three studies). Of the quantitative and mixed-methods studies, 19 studies relied on secondary data collected from service records derived from patient charts, clinic billing data, or service records of callers to safe abortion hotlines [22] or national abortion funds [28]; 14 studies relied on secondary data sources such as state or national data on abortions. Eight studies relied on data collected from the Alan Guttmacher Institute (AGI) Abortion Provider Survey of health institutions and private physicians providing abortion services [29–30, 37–41, 55]. Twenty of the quantitative or mixed-method studies relied on primary data collected via self-administered or interview-administered questionnaires with abortion clients. All of the qualitative studies relied on in-depth interviews with women seeking abortion or women who had obtained an abortion. Sample sizes ranged from 58 to 8,338 for quantitative studies that conducted primary data collection and 13 to 45 for qualitative studies.

**Table 1:** Characteristics of studies included in the review.

Study citation	Study design	Location	Sample Size	Data Source	Travel Focus
**Quantitative studies (46)**					
Aiken et al. 2016 [22]	Prospective longitudinal	Ireland	5,650 women seeking abortion; 1,023 women who obtained abortions	Service records; client surveys	TERTIARY
Brown et al. 2001 [23]	Retrospective cross-sectional	US (Texas)	146,524 white women who were pregnant in 1993; 102,185 Hispanic women who were pregnant in 1993; 42,763 black women who were pregnant in 1993	State or national data	SECONDARY
Cameron et al. 2016 [24]	Prospective longitudinal	Scotland	267 women seeking abortion	Service records	SECONDARY
Cooper et al. 2005 [25]	Prospective cross-sectional	South Africa	673 women who obtained an abortion	Client surveys	TERTIARY
Dobie et al. 1999 [26]	Retrospective cross-sectional	US (Washington)	53,287 women who had abortions between 1983–1984; 53,662 women who had abortions between 1993–1994	State or national data	SECONDARY
Ellerston 1997 [27]	Retrospective cross-sectional	US (Minnesota, Missouri, Indiana)	146,168 abortions between 1977–1990 among women aged 15–24 in Minnesota; 111,683 abortions between 1977–1990 among women aged 15–24 in Missouri; 100,512 abortions between 1978–1988 among women aged 15–24 in Indiana	Service records; state or national data	SECONDARY
Ely et al. 2017 [28]	Retrospective cross-sectional	US	3,452 women who received abortion funding; 2,716 women who received abortion funding with data on travel	Service records	PRIMARY
Forrest et al. 1979 [29]	Retrospective cross-sectional	US	744,600 abortions in 1973; 898,600 abortions in 1974; 1,034,200 abortions in 1975; 1,179,300 abortions in 1976; 1,320,300 abortions in 1977; 1,374,000 abortions in 1978	Service records	SECONDARY
Forrest et al. 1978 [30]	Retrospective cross-sectional	US	1,200,000 abortions in 1976; 1,300,000 abortions in 1977	Service records	SECONDARY
Foster & Kimport 2013 [31]	Prospective cross-sectional	US	272 women who obtained an abortion at or after 20 weeks’ gestation and 169 women who obtained a first-trimester abortion	Client surveys	TERTIARY
Francome 1992 [32]	Prospective cross-sectional	England	200 women seeking abortion	Client surveys	PRIMARY
Gerdts et al. 2016 [33]	Prospective cross-sectional	England	58 women seeking abortion	Client surveys	PRIMARY
Gerdts et al. 2016 [34]	Prospective cross-sectional	US (Texas)	398 women seeking abortion	Client surveys	PRIMARY
Grossman et al. 2013 [35]	Retrospective cross-sectional	US (Iowa)	17,956 women who had abortions	Service records; state or national data	SECONDARY
Grossman et al. 2014 [36]	Retrospective cross-sectional	US (Texas)	35,415 abortions in Nov 1 2012 –April 30 2013; 32,611 abortions in May 1 2013 –Oct 31 2013; 30,800 abortions in November 1 2013 –April 30 2014	Service records; state or national data	TERTIARY
Grossman et al. 2017 [16]	Retrospective cross-sectional	US (Texas)	66,098 abortions in 2012; 53,882 abortions in 2014	State or national data	SECONDARY
Henshaw & Finer 2003 [37]	Ecological	US	1,819 abortion facilities	Provider surveys	SECONDARY
Henshaw 1995 [38]	Ecological	US	1,492 abortion facilities	Provider surveys	SECONDARY
Henshaw et al. 1981 [39]	Ecological	US	1,400,000 abortions	Provider surveys	SECONDARY
Henshaw & O’Reilly 1983 [40]	Ecological	US	898,570 abortions in 1974; 1,316,700 abortions in 1977; 1,409,600 abortions in 1978; 1,497,670 abortions in 1979; 1,553,890 abortions in 1980	Provider surveys	SECONDARY
Henshaw 1991 [41]	Ecological	US	1,819 abortion providers	Provider surveys	SECONDARY
Jewell & Brown 2000 [42]	Ecological	US (Texas)	254 counties (state level data on abortion rates)	State or national data	SECONDARY
Johns et al. 2017 [43]	Retrospective cross-sectional	US (California)	35,431 abortions to 32,582 women	State or national data	PRIMARY
Jones et al. 2013 [44]	Prospective cross-sectional	US (Arkansas, California, Georgia, Illinois, New Jersey, and Texas)	639 women who had an abortion	Client surveys	TERTIARY
Jones & Jerman 2013 [45]	Prospective cross-sectional	US	8,338 women who obtained abortions	Client surveys	PRIMARY
Joyce et al. 2013 [46]	Retrospective cross-sectional	US	452,607 abortions in the United States in 1971; 257,857 abortions in New York in 1971; 503,423 in the United States in 1972; 277,905 in New York in 1972	State or national data	SECONDARY
Karasek et al. 2016 [47]	Prospective cross-sectional	US (Arizona)	379 women seeking abortion	Client surveys	TERTIARY
Kiley et al. 2010 [48]	Prospective cross-sectional	US (Illinois)	247 women seeking surgical abortion	Client surveys	SECONDARY
Levin et al. 2009 [49]	Prospective cross-sectional	Mexico	3,945 abortions	Client surveys	TERTIARY
Loeber & Wijsen 2008 [50]	Retrospective cross-sectional	Netherlands	254 women who obtained abortions	Service records; state or national data	TERTIARY
Lokeland et al. 2014 [51]	Prospective cross-sectional	Norway	1,018 women seeking medication abortion	Client surveys	SECONDARY
Nickson et al. 2006 [52]	Prospective cross-sectional	Australia	1,244 women seeking abortion	Client surveys	PRIMARY
Nickson et al. 2002 [53]	Ecological	Australia	73,699 Medicare claims for abortion services	State or national data	PRIMARY
Roberts et al. 2015 [14]	Retrospective cross-sectional	US (Louisiana)	5,641 women who obtained an abortion	Service records	SECONDARY
Rogers & Lenthall 1975 [54]	Cross-sectional	Australia	145 women seeking abortion	Not specified	PRIMARY
Sanders et al. 2016 [15]	Prospective and retrospective cross-sectional	US (Utah)	3,130 women seeking abortions under the 24-hour law; 3,618 seeking abortions under the 72-hour law; 307 women who obtained an abortion	Service records; client surveys	SECONDARY
Seims 1980 [55]	Ecological	US	3,105 US counties; 373,700 estimated women in need of an abortion in counties with no provider	Service records; state or national data	TERTIARY
Sethna & Doull 2013 [56]	Prospective cross-sectional	Canada	1,186 women seeking abortion	Client surveys	PRIMARY
Sethna & Doull 2007 [57]	Prospective cross-sectional	Canada	1,022 women seeking abortion	Client surveys	PRIMARY
Shelton et al. 1976 [58]	Ecological	US (Georgia)	22,000 abortions	State or national data	SECONDARY
Shochet & Trussell 2008 [59]	Prospective cross-sectional	US	205 women seeking abortion	Client surveys	TERTIARY
Silva & McNeill 2008 [60]	Ecological	New Zealand	Regional council level population data on total population and abortion rate	State or national data	SECONDARY
Upadhyay et al. 2014 [61]	Prospective longitudinal	US	452 women who had an abortion; 231 women who were denied an abortion	Client surveys	SECONDARY
Upadhyay et al. 2016 [62]	Retrospective cross-sectional	US (Ohio)	1,156 medication abortions in Jan 2010- Jan 2011; 1,627 medication abortions in Feb 2011 –Oct 2014	Service records	TERTIARY
Van Bebber et al. 2006 [63]	Prospective cross-sectional	US	212 women who received a medication abortion	Client surveys	TERTIARY
White et al. 2017 [64]	Retrospective cross-sectional	US (Alabama)	2,730 women seeking abortion; 2,216 women who obtained an abortion	Service records	PRIMARY
**Mixed methods study (1)**					
Grossman et al. 2012 [65]	Prospective cross-sectional study and in-depth interviews	US (California)	87 women seeking abortion participated in cross-sectional survey; 17 women seeking abortion participated in in-depth interviews	Client surveys Client interviews	PRIMARY
**Qualitative studies (12)**					
Baum et al. 2016 [11]	In-depth interviews	US (Texas)	20 women seeking abortion	Client interviews	SECONDARY
Cockrill & Weitz 2010 [66]	In-depth interviews	US	20 women who obtained an abortion	Client interviews	TERTIARY
Doran & Hornibrook 2014 [67]	In-depth interviews	Australia	13 women who obtained an abortion	Client interviews	SECONDARY
Doran & Hornibrook 2016 [68]	In-depth interviews	Australia	13 women who obtained an abortion	Client interviews	SECONDARY
Foster et al. 2017 [69]	In-depth interviews	Canada	33 women who obtained an abortion	Client interviews	TERTIARY
Fuentes et al. 2016 [12]	In-depth interviews	US (Texas)	23 women seeking abortion	Client interviews	SECONDARY
Grindlay et al. 2013 [70]	In-depth interviews	US (Iowa)	25 women who obtained an abortion	Client interviews	TERTIARY
Heller et al. 2016 [71]	In-depth interviews	Scotland	16 women who obtained an abortion	Client interviews	SECONDARY
Jerman et al. 2017 [13]	In-depth interviews	US (Michigan, New Mexico)	29 women seeking abortion	Client interviews	PRIMARY
Margo et al. 2016 [72]	In-depth interviews	US (South Carolina)	45 women who obtained an abortion	Client interviews	TERTIARY
Purcell et al. 2014 [73]	In-depth interviews	Scotland	23 women seeking abortion	Client interviews	TERTIARY
White et al. 2016 [74]	In-depth interviews	US (Alabama)	25 women seeking abortion	Client interviews	PRIMARY

We created a “travel focus” variable for each study in order to judge its relevance to this review, indicated in Table 1. Studies labeled “primary focus” were studies that addressed factors related to travel as a critical component to analysis. For these studies, travel was considered either a main exposure, outcome (for analytic studies), or focus (for descriptive studies). For qualitative studies with a “primary” travel focus, the instrument guide was explicitly developed to gather information about women’s experiences related to travel for abortion care. This category includes studies where the entire study population was women who traveled for abortions. Studies labeled “secondary focus” reported on results related to travel, but travel was not the primary outcome. For qualitative studies with a “secondary focus,” travel may have been mentioned in descriptions of themes, but was not an overarching theme. Studies labeled “tertiary focus” only briefly mentioned travel: no specific results were presented and travel may have only come up in the discussion. Overall, 25% of studies (15) contained travel as a primary focus, 46% (27) had a secondary focus, and 29% (17) had a tertiary focus. These patterns were broadly consistent across quantitative and qualitative studies, study location, and date of publication, although the majority of studies with travel as a primary focus (67%) were published in the last five years. Studies with travel as a primary focus were concentrated in the United States, Australia, Canada, and England; all other settings discussed travel as a secondary or tertiary focus. Among the 40 US-based studies, 20% had travel as a primary focus, 53% had travel as a secondary focus, and 27% had travel as a tertiary focus.

### Quantitative findings

Travel was measured and conceptualized in quantitative studies primarily as distance traveled and burdens related to travel, including travel costs incurred. As shown in Table 2, distance that women traveled for abortion services was measured in several ways within quantitative studies: 1) distance traveled in miles or kilometers was self-reported by patients or providers, 2) distance traveled in miles or kilometers was calculated using road networks, straight-line measurements, or geodesic formulas, or 3) time traveled was self-reported by patients. Similarly, burdens related to travel were conceptualized in quantitative studies in two principal ways. Eleven studies reported financial costs of travel, such as the cost of accommodations, gas, plane tickets, or other transportation costs. Five studies reported other burdens, including the need to arrange childcare, time away from work, and the need to disclose the abortion to others.

**Table 2:** How travel is measured and conceptualized in quantitative studies.

How travel is measured & conceptualized	Number of studies*
Distance: road network	12
Distance: straight line (Euclidean)	5
Distance: geodesic	1
Distance: patient or provider reported	10
Distance: travel time	6
Travel burdens: financial	11
Travel burdens: other (e.g. childcare, time away from work, need to disclose/ability to keep abortion confidential)	5
Self-reported out of state residency	13

*Note: Study numbers do not add up to 47 as some studies measured and conceptualized travel in multiple ways.

Quantitative studies (including one mixed-methods study) in this review considered a domain of travel as an outcome of interest (37 studies) or as an exposure (11 studies); one study explored travel as both an outcome and exposure [47]. Table 3 displays the range of approaches that studies took to understanding travel as a component of abortion seeking, often as a main outcome of a study that considered how far women needed to travel for abortion services [28, 43, 45, 65] and sometimes as a secondary exposure, as in studies around home administration of medical abortion [51], abortion provider preference [59], or provider availability and abortion demand [23]. Many studies examined travel by measuring the amount of women who traveled out of state for abortion services [28–30, 39–40, 53].

**Table 3:** Characteristics related to travel among quantitative studies in the review.

Study citation	Exposure	Outcome	Main Question Related To Travel	How travel measured/conceptualized	Main conclusion
Aiken et al. 2016 [22]	Descriptive	Demographic characteristics, reasons for seeking at-home medical termination of pregnancy	What are women's reasons for seeking at-home medical termination of pregnancy	Self-reported descriptions of experiences seeking at-home medical termination of pregnancy	Women who used Women on Web's telemedicine service reported in open-ended questions barriers to traveling abroad for abortion services including: cost of travel, arranging childcare, taking time off work, needing to disclose travel to family members, and migrant status.
Brown et al. 2001 [23]	Travel cost	Probability of a pregnancy ending in abortion	Responsiveness of abortion demand to variations in the travel-cost component of the full cost of abortion services	Travel cost calculated as travel distance from center of woman's country of residence to nearest city with abortion services.	Pregnant women who reside in counties with longer travel distances to the nearest abortion providers have lower probabilities of aborting their pregnancies than women in counties closer to abortion providers. Simulations show that changes in travel distances will have relatively large impacts on overall abortion rates and these effects vary across race. A 10% decrease in travel distance would result in a 2.37% increase in the probability of a pregnancy ending in abortion for white women, 5.36% increase for Hispanic women, and 2.79% increase for black women.
Cameron et al. 2016 [24]	Descriptive; gestational age	Traveling for abortion	What are the characteristics of women presenting at ≥ 16 weeks gestation for abortion in Scotland?	Women who presented at Scotland facilities beyond the local gestational limit but whose pregnancies ended in abortion (assumption is that they needed to travel to procure the abortion)	Of the 267 women, 18.7% (50) proceeded to abortion by traveling to England. Women who presented beyond 20 weeks had 6.37 times higher odds of continuing the pregnancy than those who presented in the 16th week (all women above 20 weeks would have had to travel for abortion services).
Cooper et al. 2005 [25]	Province	Travel distance, mode, and cost	What are women's experiences traveling for abortion services, and how does it differ by province?	Travel time (dichotomized <1 hr., > = 1 hr), distance travelled in kilometers (0–10, 11–50, 50–100, > 100), mode of transport (own, public transport, walk other), and travel cost (in South African Rand)	Most women (58%) and especially urban women (64%) traveled ≤ 10km to reach health facilities. Travel distances were longer for rural women: 66% travelled >10km and 16% travelled >50km to reach a health facility. Most women used public transportation. 86% of urban women travelled for < 1 hr, while 59% of rural women traveled for more than 1 hour. Rural women paid more for transportation.
Dobie et al. 1999 [26]	Time period (1983–1984 vs. 1993–1994)	Travel distance to abortion clinic	Change in travel distance to abortion clinic from 1983–1984 and1993-1994	Using state level data, calculated one way distance in miles between the abortion patient’s resident and the location of the provider	Rural women traveled farther for an abortion in 1993–1994 than in 1983–1984, and this difference was greater among older women.
Ellerston 1997 [27]	Before and after the implementation of parental involvement laws	Traveling for abortion services	Impact of parental involvement laws on minors' travel to other states for abortion services	Amount of in-state abortions and abortions in neighboring states	The odds of travel increased for minors by over 50% when the law took effect. Increases for older teenagers and women in their early 20s were significantly smaller, at 13% and 18%.
Ely et al. 2017 [28]	Insurance coverage, region/state of residence, gestational age	Distance traveled, likelihood of out of state travel	Effect of various characteristics on the distance women travel for abortion services and likelihood of out of state travel	Kilometers traveled from residential zip code to clinic, state of residence and state where abortion was expected to be performed	Women in states where private insurance restricts abortion coverage traveled farther distances. 31.2% of women traveled out of state for abortions and those who traveled out of state traveled 10 times the distance as those who did not. Traveling out of state was more likely for women in non-expanded Medicaid states and those in the second trimester. Travel out of state was less likely for women in Midwestern states and those with restrictive private insurance. Average travel distance increased over 100 km from 2010 to 2014.
Forrest et al. 1979 [29]	State of residence	Out of state travel	Proportion of women traveling out of state for abortion services	Number of abortions of nonresidents by state	9% of women traveled to another state for abortion services. More than half the residents of 6 states who received abortions traveled to another state. States' provision to nonresidents varies widely- some states are places women go for services and some are places women leave for services elsewhere. NY, DC & WA provided the most abortions to nonresident women.
Forrest et al. 1978 [30]	State of residence	Out of state travel	Proportion of women traveling out of state for abortion services	Number of abortions of nonresidents by state	4 out of 10 women traveled outside their home counties to obtain abortions and 10% traveled outside of their state. More than 40% of the residents of 4 states who received abortions traveled to another state. States' provision to nonresidents varies widely- some states are places women go for services and some are places women leave for services elsewhere. NY, DC & CA provided the most abortions to nonresident women.
Foster & Kimport 2013 [31]	Women who had abortions after 20 weeks vs. women who had abortions in the first trimester	Demographics, abortion characteristics, delays	Differences in travel experiences to clinic based on gestational age	Travel time to clinic (dichotomized as less than or equal to 3 hours or more than 3 hours), open ended responses to delays in reaching care related to travel categorized under "difficulty getting to the abortion facility" and "raising money for procedure and related costs"	Women who obtained abortions after 20 weeks were more likely to have traveled more than 3 hours to reach a clinic (21% vs. 5%). Women who obtained abortions after 20 weeks were more than twice as likely than first trimester patients to report that difficult getting to the abortion facility slowed them down (27% vs. 12%) and spent more on transportation to the abortion facility.
Francome 1992 [32]	Descriptive; entire population is traveling women	Demographics, birth control usage	Characteristics of women who travel from Ireland to England for abortion services	Irish women obtaining abortions in Marie Stopes clinics in England	Women from Ireland traveling to England for abortion tended to discuss their birth control options and decision around their pregnancy with their boyfriend or husband. 32% of participants were not using a form of contraception when they became pregnant. No specific information on their travel experiences provided.
Gerdts et al. 2016 [33]	Descriptive, entire population is traveling women	Experiences related to travel	Travel experiences of women who travel to England for abortions	Burden of travel cost, taking time off work, taking time away from caretaking, length of stay in England, method of travel, traveling alone, travel cost, overnight stay, cost of accommodation, type of accommodation	Women commonly reported reasons for traveling for abortion as abortion was not legal (51%), followed by having passed the gestational limit for a legal abortion (31%). Women paid an average of £631 for travel expenses, and an average of £210 for accommodation. 50% reported that it was difficult or very difficult to cover the cost of travel.
Gerdts et al. 2016 [34]	Clinic closures, restrictive laws	Distance traveled	Impact of clinic closures on the distance women travel for abortion services	Road miles from zip code of residence to nearest open clinic and clinic where woman was interviewed taken at two time points (2013 & 2014)	Total population's distance from zip code of residence to nearest open clinic increased after TRAP laws were passed (mean 20 mi). Women whose nearest clinic closed in 2014 traveled an average of 52 miles more to reach their nearest open clinic in 2014 than they did in 2013. Clinic closures resulting from TRAP laws increased travel distance four-fold for women whose nearest clinic closed and for 44% of this group, the new distance exceeded 50 miles.
Grossman et al. 2013 [35]	Telemedicine	Distance traveled, distance of patient to nearest clinic that offered surgical abortion	Impact of telemedicine on distance traveled to an abortion clinic	Straight-line calculations between clinic and patient residential zip codes, US census shape files/zip code areas used to create spatial clusters	After telemedicine was introduced, distance traveled decreased, access for women living in remote areas increased (number of women that lived farther away accessing services increased), and medical abortion patients were more likely to live 50+ miles from surgical abortion clinic. Medical abortions increased among patients living 50+ miles from a surgical abortion clinic.
Grossman et al. 2014 [36]	Before, during, and after debate and passing of HB2	Distance to nearest abortion provider	Impact of clinic closures on women's travel distance to the nearest abortion provider	Computed travel distance from 4 metropolitan areas (Austin, Ft. Worth/Dallas, San Antonio, Houston) to the nearest Texas county in which there was at least one abortion provider, using Traveltime3 in Stata version 13.0, which accesses the Google Distance Matrix Application Programming Interface.	Approximately 10,000 women in the 6 months before the debate and passing of HB2 lived >200 miles from a Texas clinic providing abortions; this increased to 290,000 after HB2 restrictions began to be enforced. More than twice that many woman will live >200 miles from a Texas clinic when the ambulatory surgical center requirement goes into effect.
Grossman et al. 2017 [16]	Distance to nearest facility	Number (decline) of abortions	Impact of travel distance to nearest abortion facility on the abortion rate	Distance from centroid of county to nearest open facility	Declines in abortions increased as distance to nearest facility increased.
Grossman et al. 2012 [65]	City/state of residence in Mexico; entire population is traveling women	Gestational age, reproductive health knowledge, reason for traveling for abortion, barriers to travel	Experiences of Mexican women traveling to San Diego for abortion services	City/state of residence, travel costs (transport, child care, time off work)	6% of women in overall clinic sample traveled from Mexico. Barriers to travel to US from Mexico for abortion are informational, logistical, and financial, including transportation costs, passport/paperwork, and clinic information or where to go.
Henshaw & Finer 2003 [37]	Geographic region, facility type, provider size	Distance traveled	Travel distance to abortion services	Miles from residence to facility	25% of women using nonhospital facilities traveled 50 or more miles for services, women in East South Central and West North Central traveled farther than other regions, women who used large providers (1000+ clients) were most likely to travel long distances
Henshaw 1995 [38]	Geographic region (census division), facility type, provider size	Distance traveled	Travel distance to abortion services	Miles from residence to facility (estimated by provider)	24% of women using nonhospital facilities travel 50 or more miles for services, women in East South Central region travel farther than other regions, women who use large providers (1000+ clients) most likely to travel long distances
Henshaw et al. 1981 [39]	Descriptive; state of residence	Obtaining abortions out of state	Percentage of women who travel out of state for abortion services	Needing to obtain abortion services outside of state of residence	Overall, 8% of abortions occurred outside of the woman's state of residence. In the five states with the fewest abortion facilities, around half of women obtaining abortions went out of state, compared to the five states with the most adequate abortion services, in which on average 4% of women traveled out of state to obtain abortions.
Henshaw & O’Reilly 1983 [40]	Descriptive; state of residence	Obtaining abortions out of state	Percentage of women who travel for abortion services	Needing to obtain abortion services outside of state or country of residence	In the 14 states with county data available, 8.4 percent of abortions were obtained by women outside their state of residence, and 27.2 percent by women in their home state but outside their county of residence. Overall, 7% of abortions occurred outside of the woman's state of residence, with a higher proportion in states with inadequate abortion facilities. Women who needed to travel outside of their county for first-trimester abortion services were at gestational ages 4.7 days greater than those who did not need to travel.
Henshaw 1991 [41]	Descriptive	Distance traveled	Percentage of women who travel out of state for abortion services	Providers estimated proportion of patients traveling 50–100 miles and those traveling more than 100 miles to reach the facility	An estimated 27% of nonhospital abortion patients in the United States traveled at least 50 miles from their homes to reach the clinic; 18% traveled 50–100 miles and 9% made over a 100 mile trip.
Jewell & Brown 2000 [42]	Descriptive; travel cost	Abortion rate	Responsiveness of abortion rate to variations in travel cost, and differences in travel cost by county	Travel cost estimated as the travel distance in road miles from center of each county to nearest city with an abortion provider times county median income per minute	Mean travel cost in a county that currently has abortion providers is $3.61 and $15.48 in counties without abortion providers. In their model, travel cost is negatively associated with the abortion rate (measured both per woman and per pregnancy). Overall mean travel cost is $14.59, a $1.00 increase in travel cost results in a .86% decrease in abortion rate per woman and .67% decrease in the abortion rate per pregnancy.
Johns et al. 2017 [43]	City/state/zip code of residence	Distance traveled	Characteristics of women related to travel	Miles traveled and travel time via road to provider	Mean travel distance was 23.5 miles and 12% traveled more than 50 miles. Teens, Hispanic and Asian women, and women seeking medication abortion were less likely to travel 50+ miles. Women obtaining second trimester or later abortion, obtaining hospital-based services, and rural women were more likely to travel 50+ miles.
Jones et al. 2013 [44]	Gestational age, insurance coverage	Travel costs	Travel costs experienced by women	Travel costs including hotel	6% of participants reported travel costs including hotel. More second trimester patients (15.4%) reported travel costs than first trimester patients (3.7%)
Jones & Jerman 2013 [45]	State of residence, local TRAP laws (waiting period), region of residence, SES, age, race	Distance traveled	Factors associated with distance traveled	Miles traveled from zip code of residence to clinic	Women were more likely to travel longer distances (100+ miles) if they: 1) live in a rural area, 2) live in a state with waiting period laws, 3) have higher levels of education, 4) have higher gestational age, or 5) are white.
Joyce et al. 2013 [46]	Distance to abortion provider in New York, year, abortion law, insured unemployment rate, per capita income, the percent of the female population that was nonwhite, contraception availability	Abortion rate	Identify the effect of distance to a legal abortion provider on abortion rates	Average travel distance by state measured as averaging the county-level straight line distances to nearest abortion provider weighted by the county-level population of women 15–44 years of age	The overall abortion rate fell by 1.02 abortions per 1000 women 15–44 years of age when distance increased from 183 to 283 miles prior to Roe. Distance to nearest legal abortion provider by four state groupings between 1972 and 1973 decreased from 521 miles on average to 29 miles in the post-Roe period.
Karasek et al. 2016 [47]	SES (poverty status); travel distance	Travel costs, distance traveled / delay of paying other expenses, delays in care, perceptions of delays	Women's perceptions of the impact of a waiting period law on their experiences	Distance to clinic in miles, travel costs in dollars and minutes, expenses in travel time, staying overnight, transportation	Mean travel distance was 58 miles, and 10% traveled more than 2 hours. Income differences were significant for delays in getting care because of travel costs. Travel time was greater for women below the Federal Poverty Level and they paid more in travel costs, including transportation and overnight stay.
Kiley et al. 2010 [48]	Distance traveled	Gestational age	Factors associated with abortion in the second trimester	Miles from where the participant lived to the clinic, categorized as <20 mi, 21–80 mi, >81 mi (short, medium, long distances)	10% of women had problems getting transportation to the clinic, with second trimester patients identifying this more often than first trimester patients. Second trimester patients were more likely to travel long distances (>81 mi) to obtain services.
Levin et al. 2009 [49]	Facility and type of procedure	Abortion cost	Cost implications of unsafe abortion	Abortion cost includes estimated patient cost of travel (not specified how this is measured)	The average cost per abortion with dilatation and curettage was US $143; manual vacuum aspiration was US $111 in three public hospitals and US $53 at a private clinic. The average cost of medical abortion with misoprostol alone was US $79. The average cost of treating severe abortion complications at public hospitals ranged from US $601 to over US $2,100.
Loeber & Wijsen 2008 [50]	Non-resident status	Second trimester abortion	Factors associated with abortion in the second trimester	Number of women who traveled to the Netherlands for abortion	36% of the sample traveled from abroad. A very high proportion of women from abroad obtained second trimester abortions. Women who traveled for abortions reported delays in their country of origin and needing more time and/or money for the procedure.
Lokeland et al. 2014 [51]	Travel time to abortion provider	Level of pain, bleeding, need for surgery, and acceptability	Compare acceptability and efficacy of medical abortion at home by women's distance to abortion clinic	Travel time by car calculated using address of patient’s residence and clinic on the “Visveg” website managed by the Norwegian Public Roads Administration: women categorized as living 60 minutes or closer in distance compared to more than 60 minutes away.	Distance to the clinic had no impact on the acceptability of home administration of misoprostol or on treatment outcome variables.
Nickson et al. 2006 [52]	Age, state/postcode of residence	Distance traveled, travel costs	Travel experiences of women who access private abortion services in Victoria	Kilometers traveled from postcode to clinic, travel time in hours, travel cost (increments from <$10 to >$100), overnight accommodation need	9.3% of women traveled more than 100km to clinic. 35% of women spent over 1 hour traveling to clinic. 12.1% spent over $50 on travel costs. 7.3% needed overnight accommodation. Younger women were more likely to travel long distances and spend more time and/or money traveling. Teenagers were 2.5 times more likely to travel more than 100km and 3.5 times more likely to spend 3+ hours traveling. Aboriginal or Torres Straight Islander women were 5.1 times more likely to travel more than 100km than non-ATSI women.
Nickson et al. 2002 [53]	State/territory of residence	Out of state travel	Utilization of interstate abortion services	Medicare claims for abortion services made by residents and nonresidents by state	Based on location of Medicare claims and applicants' state of residence, women are traveling between states for abortion services.
Roberts et al. 2015 [14]	Clinic closure	Travel distance	Impact of clinic closures on travel distance to abortion provider	Woman's zip code of residence and location of facility where she received abortion care; projected travel distance after clinic closure calculated by measuring distance from closest "open" clinic from parish centroid to provider for each parish.	Women who had abortions in Louisiana traveled a mean of 71 miles each way for their abortion, with Lousiana residents traveling a mean distance of 51 miles each way. If all Louisiana facilities close, the mean distance women would need to travel would more than triple to 208 miles; 100% of the abortion patients who reside in Louisiana would have needed to travel at least 50 miles, and 76% would need to travel more than 150 miles each way. The proportion of Louisiana women of reproductive age who live more than 150 miles from an abortion facility would increase from 1% to 72%.
Rogers & Lenthall 1975 [54]	Descriptive; entire population is traveling women	Demographics, abortion characteristics, cost	Experiences of women from New Zealand who travel to Australia for abortion services	Women who obtained abortion services in Melbourne that were from New Zealand	Women spent $450 for flights, accommodation, and the abortion procedure; travel costs prevented patients of all ages from being accompanied by parents, boyfriends or husbands.
Sanders et al. 2016 [15]	HB 461 (72 hour wait time law) enacted	Lost wages, childcare costs, school days missed, distance traveled for counseling, distance traveled for abortion procedure, transportation costs, and the ability to keep their abortion confidential	Self reported distance traveled for counseling and the procedure, transportation costs	Impact of wait time on travel experiences	About two-thirds of women could be counseled and consented within 25 miles from their home; however, only 42% could receive their abortion procedure within 25 miles. More than 10% of women traveled more than 100 miles from their home for their procedure. A substantial number of women reported lost wages (47%), excess childcare cost (18%), increased transportation cost (30%), and additional expenditures and lost wages by a family member or friend (27%).
Seims 1980 [55]	Descriptive; state	Proportion of counties with no abortion provider, unmet need	Presence/absence of provider, proportion of women who obtain abortions in their county of residence	Areas in the United States were abortion is inaccessible	The women who experience perhaps the greatest difficulty in locating abortion services are those who live not only in a county without a provider but also in one that does not border a county containing a facility to which thy might travel to obtain an abortion. There were an estimated 195,100 women in need of abortion services who would probably have had to travel some distance from their home communities for abortion services.
Sethna & Doull 2013 [56]	Descriptive	Distance traveled to clinic, travel experiences	Spatial disparities in access to abortion for women in Canada	Self-reported distance from home to clinic, transportation costs, childcare costs, lost wages, ease of travel	Women living in Canada's rural, Northern and coastal communities are underserved.
Sethna & Doull 2007 [57]	Descriptive; age, income	Distance traveled to clinic, travel experiences	Women's experiences traveling for abortion services	Self-reported distance from home to clinic, mode of travel, travel time, transportation costs, ease of travel	Majority of women (73.5%) traveled an hour or more to the clinic. Slightly more than 15% of women traveled between 100km and 1000km to get to the clinic. Most women traveled by car with a companion which increased cost for overnight accommodations.
Shelton et al. 1976 [58]	Travel distance to Atlanta	Abortion ratio	Effect of travel distance on abortion utilization	Highway distance from home county to Atlanta	Distance to Atlanta is negatively correlated with the abortion rate in each county; the correlation between distance and utilization appears to be stronger for black women than for white women.
Shochet & Trussell 2008 [59]	Travel time	Preference for own OB-GYN or regular physician as provider for abortion services	Effect of travel on preference for provider	Participant-reported one way travel time to clinic, measured in minutes.	Women who would have preferred their OB/GYN were more likely to have spent a longer amount of time traveling to the clinic. Travel time was not a predictor of preferring one's regular physician for the abortion services.
Silva & McNeill 2008 [60]	Descriptive; Regional Council Area	Distance to nearest abortion provider	Compare distance traveled for abortion care across regions	Round-trip travel distance between abortion client's residence and clinic location where abortion was obtained calculated with online driving distance calculator	Women who live in regions that do not offer local termination of pregnancy (TOP) services travel on average 442km round trip to access TOP services. Three of the five regions that do not have local TOP services available have a higher than average proportion of Maori population.
Upadhyay et al. 2014 [61]	First trimester vs. Near-limits; Near-limits vs. Turnaways	Distance to provider, delays by travel and procedure costs	Compare distance to provider and delays associated with travel between	Distance between participant and provider zip codes calculated by Stata, participant reported delays	For near-limits and turnaways, the most common delay was travel and procedure costs; these costs were higher for turnaways compared to those who obtained abortions in the first trimester. Near-limits traveled farther to reach a clinic than those who terminated in the first trimester and those who were turned away.
Upadhyay et al. 2016 [62]	Pre-law period and post law period	Distance traveled to abortion clinic	Effect of law on travel	Distance traveled to abortion care calculated based on home zip code to facility using the “traveltime3” Stata module	Most women (86%) traveled <50 miles for abortion care, and 13% travelled 50 miles or more. Pre and post-law populations did not differ by travel time, though women who traveled more than 50 miles were less likely to return for a follow-up visit.
Van Bebber et al. 2006 [63]	Descriptive	Travel costs	Describe travel costs associated with abortion	Patient reporting of costs for transportation and accommodation	44% of women reported travel expenses over $0. Of those who reported transportation expenses, the mean cost was $18 with a range of $1 - $100.
White et al. 2017 [64]	Travel distance	Whether women returned for abortion procedure, number of days between consultation and procedure visit	Association between travel distance and returning for abortion procedure	Distance traveled between woman’s residential zip code and the facility where she attended the consultation visit, calculated using Stata’s traveltime3 command	58% of women traveled less than 25 miles one way to the clinic, 13% traveled 25 to 49 miles, 21% traveled 50 to 100 miles, and 8% traveled more than 100 miles. Overall, 19% of women did not return to a clinic for an abortion procedure after their consultation. Distance traveled was not associated with return for an abortion visit.

Studies that relied on self-administered or interview-administered surveys with abortion clients tended to focus explicitly on women’s experiences related to travel for abortion care and conceptualized travel as the distance to the clinic along with additional travel-related burdens such as transportation and accommodation costs, time, and impacts on delays to care. These studies were all convenience samples that recruited participants from abortion clinics or provider settings. Studies that relied on state or national data were primarily focused on modeling the distance between counties of residence and the nearest abortion provider or the number of women who travel across state or national lines for abortion services. These studies relied on national or state surveillance reports from the study time period, or data on the number of abortions reported by the AGI Abortion Provider Survey, a comprehensive survey of all known abortion providers in the United States. Studies that relied on service records were mainly focused on the straight-line distance that clients traveled for their service and the number of out of state clients. These studies used all eligible records from a specified time frame (usually a particular year or range of years).

Findings from studies included in this review suggest that the limited availability of abortion providers, insurance restrictions, as well as gestational age and other legal restrictions result in women needing to travel long distances for abortion services, often crossing state or country borders to seek care [27, 28, 31, 33, 45, 50, 60]. Studies that rely on aggregated county-level data on the number of abortions found that abortion rates are lowest in counties that are farther from counties with abortion providers or have no abortion provider [16, 23, 42, 46, 58]. Studies that relied on state-level or national data described the phenomenon of women needing to travel out of state or to a different province for abortion services [28–30, 39, 40, 53] or to a different country [50, 24]. Studies describe the substantial distances that women often need to travel in order to obtain abortion services; in these studies, many participants traveled over 50, 100, or even 200 miles to reach services [14, 15, 25, 26, 34–38, 41, 43, 47, 48, 52, 55, 57, 60, 62, 64]; rural women [25, 26, 43, 45], women with gestational ages over 12 weeks [43, 45, 48], younger women [52], and women of lower socioeconomic status [47] were more likely to have to travel longer distances.

Studies describe a variety of additional burdens that traveling for abortion services places on women. Participants in these studies explicitly cite the cost of travel expenses [15, 22, 33, 44, 47, 52, 54, 63, 65], which include the cost of transportation, accommodation, childcare expenses, and lost wages as a barrier to reaching timely care when needing to travel for services. Some studies found that these burdens related to travel significantly negatively impacted participants’ reproductive choices; needing to travel long distances for abortion services, particularly the logistical challenges and financial burdens associated with arranging travel, delayed women from accessing care in the first trimester [24, 31, 40, 61], or prevented them from being able to obtain an abortion at all [24, 61]. Two studies demonstrated the potential of telemedicine or at-home administration of medication abortion to reduce the burdens of traveling for services and increase access, particularly for women who live far away from an abortion provider [35, 51].

### Qualitative findings

**Fig 2:**
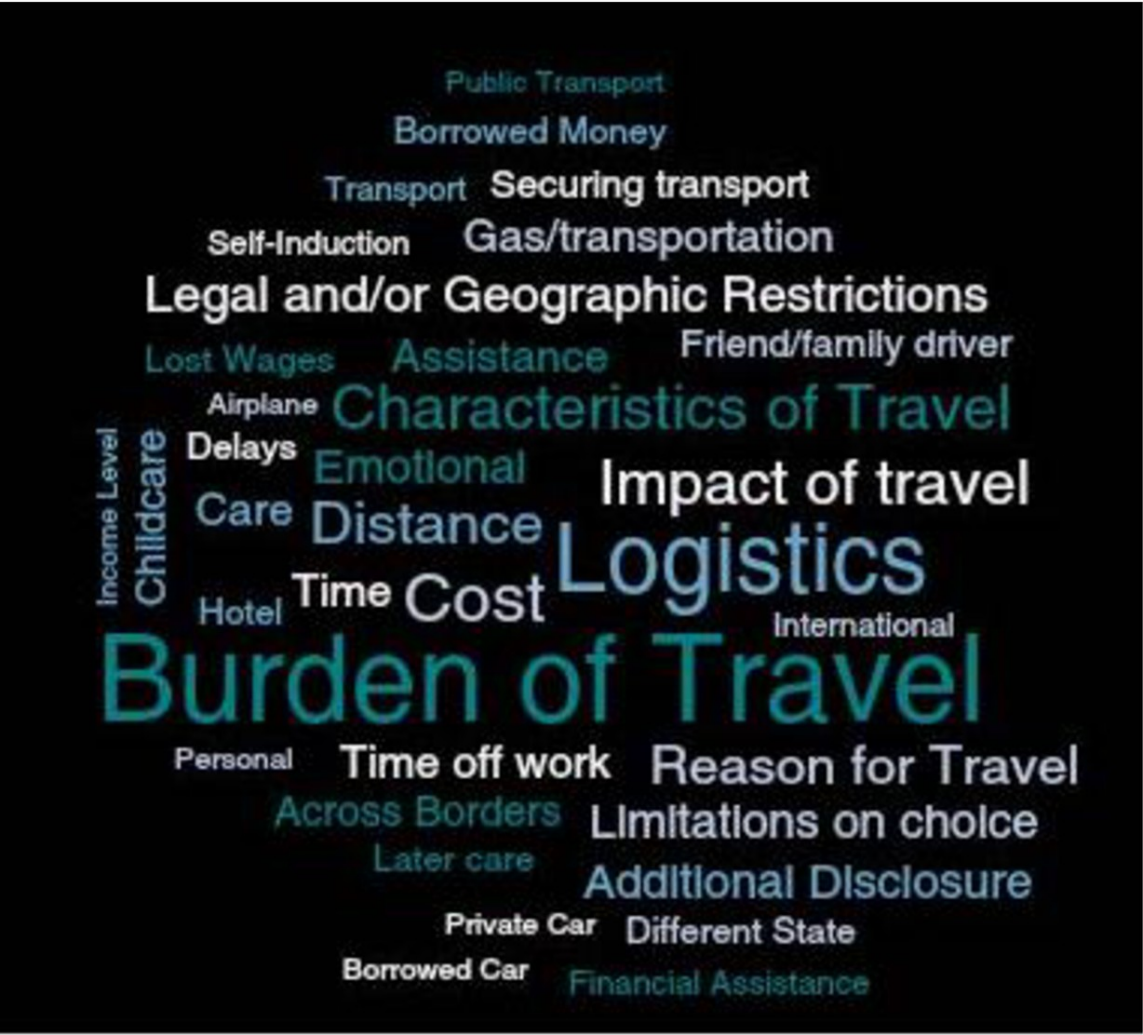
Travel-related themes in qualitative studies.

All qualitative studies relied on in-depth interviews with women, either in person or via telephone/Skype, as their source of data. Of the twelve qualitative studies included in this review, two had travel as a primary focus [13, 74], five considered travel as a secondary focus [11, 12, 67–68, 71], and five considered travel as a tertiary focus [65, 69–70, 72–73]. All studies described women traveling either within or to countries where abortion is ostensibly legal albeit restricted (the United States, Scotland, Australia, and Canada); the context of many of these papers are to explore how laws or regional disparities in access affect women’s experiences with abortion care, including their need to travel for services. Broad themes that emerged in how travel was conceptualized or discussed were the descriptive characteristics of travel, the burdens that needing to travel for abortion services imposed on women, the impact of travel on women’s care and abortion method choices, and the reasons for travel. Key findings are presented below and themes from the qualitative studies, represented by commonly-seen terms, are presented in Fig 2. Codebooks can be found in Appendix 6.

#### Characteristics of travel

Almost all studies in this review contained descriptions of the modes of transportation women used when traveling for abortion services. Participants described traveling for abortion via airplane, private car, and public transportation. Needing assistance to arrange travel or obtain transport was described by participants in eight studies; for example, participants described needing to borrow money, borrow a car, or rely on a friend or family member for transportation to their appointments. Descriptions of characteristics of travel most commonly emerged in studies where travel was a secondary or tertiary focus.

#### Burdens of travel

Travel as a barrier to abortion care emerged as a theme in all studies, regardless of the travel focus of the study. Sub-themes related to the burdens of travel included logistical burdens, emotional burdens, cost, and time. Logistical burdens entailed the physical distance that women traveled to reach abortion providers, securing transportation, making childcare arrangements, and obtaining time off from work. Emotional burdens that were a direct result of travel included feeling uncomfortable and lonely while traveling alone for a procedure, feeling stressed from the need to figure out transportation and other logistics, as well as feeling stigmatized for the need to travel for routine medical care. Costs related to travel, including gas, hotel, childcare, and travel time, were mentioned in almost all studies.

In all but two studies, disclosure of having or needing an abortion, often to individuals that participants did not want to tell, were a direct result of the burden posed by needing to travel. Participants discussed how the need to secure time off of work, arrange childcare, or borrow money for travel or the procedure necessitated disclosing their decision to have an abortion to people at work and in their personal lives.

#### Impact of travel burdens on care

The impact of travel on women’s abortion care was discussed in all but two studies. Most studies focused on the impact that travel had on women’s choice of abortion method, obtaining care later than the woman would have wanted, and women’s consideration of self-inducing. All but three studies discussed the impact of travel on restricting women’s choice around their preferred method of abortion. For example, studies described how women chose surgical abortion over medication abortion because it would limit the distance they would need to travel to the clinic, the number of visits, as well as the possibility of experiencing abortion symptoms while traveling. In addition, studies also described how the burdens of travel necessitated staying overnight in order to facilitate their chosen method. In addition to limitations on method choice, the logistical aspects of travel described above often delayed women from getting to the clinic, resulting in restrictions to care based on gestational age. Four studies described how burdens imposed by travel were so great that they forced some participants to consider or attempt self-inducing to end their pregnancies.

#### Reasons for travel

Women’s stated reasons for travel were often not explicitly addressed in the included studies. When stated, almost all reasons were framed in the contexts of increased legal restrictions that limited women’s access to clinics or where residence in regions in which legal barriers to care necessitated travel, including presenting beyond gestational age limits for termination. Personal reasons outside of imposed restrictions rarely emerged; in one study, women reported traveling to the United States for abortion care because of perceived lack of safety of the procedure in Mexico (their country of residence) [65].

### Critical appraisal

Studies were critically appraised using the STROBE checklist for quantitative studies [19], the CASP checklist for qualitative studies [20], and the MMAT checklist for mixed methods studies [21]. Each checklist assessed methodological quality of studies, including sampling, analysis, and bias considerations. Overall, study quality was good, and most studies adhered to principles of rigorous study design and analysis based on the checklist criteria. Older studies provided less information about recruitment, sampling, and data analysis techniques, resulting in better quality scores for studies published in the last 20 years.

For quantitative studies, quality issues arose around two main areas: participant flow and acknowledging bias within study design and results. Participant flow within studies was the largest area for improvement in reviewed studies. Many studies did not report numbers of participants at each stage of study (26 studies, 55%), explain how missing data was addressed (27 studies, 57%), or give reasons for non-participation at each stage (36 studies, 77%). Reporting on participant flow may help to ensure methodological quality as selection bias, missing data, and loss to follow-up can impact results, especially when discussing potentially sensitive topics around abortion experiences. 28% of studies did not acknowledge potential sources of bias in their studies, including possible bias within study design and the interpretation of results. Some studies may have worked to limit bias and did not report this; exhibiting transparency in these efforts would have resulted in higher critical appraisal quality scores. Older studies showed lower quality in these areas which may indicate a trend of acknowledging limitations with regard to study results; however, a majority of studies (68%) did not consider bias in study design, indicating room for improvement.

In qualitative studies, the principal quality issue involved a lack of consideration of the relationship between researchers and participants and the researcher’s examination of their own role, biases and influence during formulation of the research question and data collection (57% of studies). Ethical issues and recruitment strategies were also areas for improvement, including details of how research was explained to participants, if ethics committee approval was sought, and if discussion around recruitment involving why participants were the most appropriate for these studies and why some participants did not choose to take part in the study took place.

## Discussion

Given the different ways that travel has been measured and conceptualized in the literature, it is difficult to provide a cohesive summary statement of the conclusions of the studies in this review. However, taken together, these studies paint a picture of the reasons why women travel for services, barriers and delays they experience in traveling, the impact of this travel on their lives and reproductive choices, as well as possible solutions for reducing or eliminating this burden. Studies in this review suggest overall negative outcomes related to travel for abortion, including barriers related to monetary travel costs, time away from work, the need for childcare, and the need to disclose the abortion to others. Vulnerable populations were often more affected by travel, with younger women (including teenagers), women of color, and rural women traveling farther distances to access abortion services. Gestational age played a role as both an exposure and outcome related to travel in the reviewed studies: women at higher gestational ages often traveled farther distances to access abortion, and women whose limited access to abortion necessitated farther travel distances experienced delays that resulted in higher gestational ages or prevented them from obtaining an abortion altogether. Overall, travel impacted on women’s access to abortion in multiple ways; causing delays, monetary costs, and other burdens.

Understanding and measuring the experience of traveling for abortion services is a complex challenge. A simple measurement of distance traveled will not suffice to capture the personal impact that distance has on the individual seeking an abortion. For example, if a woman does not have access to a personal car, then she is more likely to need to involve others in order to secure transportation, regardless of whether the distance is 15 miles or 50 miles. Similarly, there are relative measures of cost and additional burdens that women experience as a result of their travel for abortion services, some of which can be measured quantitatively and others qualitatively. While travel was approached in a range of ways and with a varying level of focus across the studies in our review, it was notable that there was no consistent measure for abortion related travel. This measurement inconsistency makes it difficult to compare travel outcomes across studies. Even a straightforward measurement like distance traveled was calculated by study authors using three different types of mathematical concepts. No pattern for use of travel measurement type (i.e. distance traveled by straight line, distance traveled by road network, time traveled) existed across studies: studies with travel as a primary, secondary, and tertiary focus showed equal amounts of each measurement.

Inconsistent measurement of travel is not unique to abortion related studies; the studies in this review reflect the wider healthcare field in terms of the variety of travel distance measurements used [75]. Standardizing measurements for abortion related travel, however, could have very real implications for policy planning and litigation, and researchers focusing on abortion should make efforts to address this issue. Standardized measures for travel related to abortion should be widely implemented to give an accurate, generalizable picture of the burden of travel for abortion on women’s lives. The WHO recommends using travel time, rather than travel distance, as a measure of accessibility [76]; only six out of the 47 quantitative and mixed methods studies in this review used this measurement. Straight distance (Euclidean) and geodesic distance measurements may not accurately capture distance traveled, especially for women in rural areas who are often the target population in studies focusing on abortion. Recent studies, including 12 studies in this review, have started using road distance; with the increasingly worldwide coverage of Google Maps, researchers can use commands within Stata and similar software like Redivis to more easily estimate road distance and travel time measurements. Researchers may also look to recent real-world examples to create standardized measures of reasonable distances to travel for healthcare services; for example, the Veterans Access, Choice, and Accessibility Act of 2014, recently extended in 2017, allows veterans located farther than 40 miles (road distance) from the closest VA facility to seek care elsewhere [77].

Similarly, the variable of financial travel cost was not standardized in the reviewed studies. Financial costs were categorized in a variety of ways (e.g. $5–10, under $50, under $100). Existing national US data on out-of-pocket financial costs for abortion services [78] could be used to develop standardized cost measurements that would allow for comparison across studies in the United States and beyond. Financial costs for travel were rarely presented in the context of local, national, or individual expenses; national data on out-of-pocket health care expenses could be used as a standardized measurement of comparison to give context to women’s financial burden of travel. In the United States, this data is measured in the Medical Expenditure Panel Survey as 1) the percentage of people with health expenses that had out of pocket expenses, 2) out of pocket expenses as a percentage of overall expenses, and 3) the average out of pocket expense per person [79]. Other burdens related to travel, such as time away from work and the inability to keep one’s abortion confidential, were reported more often in qualitative studies. Qualitative research distinctly provides an opportunity to center the individual’s experience of travel and to illustrate the complexity of experiences traveling for abortion services, and the interdependency of burdens in a way that quantitative instruments are not able to measure. More research should be done in this area to explore and accurately capture different dimensions of the relative burden of travel on women’s lives. Additionally, qualitative results on other burdens of travel, such as forced disclosure and impact on decision-making, should be used to inform quantitative research, including data collection instruments. Most of the quantitative studies in this review relied on service records, or state/national data; as a result, analyses were often limited to calculating travel distance between clients’ counties of residence and the location of the nearest clinic or provider. Future studies that directly survey women on their experiences related to travel may allow for a more nuanced and complete picture of the burdens related to traveling for abortion services to be documented in the published literature.

The majority of the studies in this review (40) are US-focused. There are several key differences between the United States and low- and middle-income countries (LMICs) in terms of travel distance and out-of-pocket cost for healthcare, including abortion care. Legal landscapes in Sub-Saharan Africa, the Middle East, Asia, and Europe often make travel for abortion necessary and unsafe abortion more commonly practiced [1]. Women in Sub-Saharan Africa often must travel long distances for all reproductive healthcare services, and out-of-pocket costs often prevent them from seeking care [80]. Despite some potential similarities in the need to travel for abortion care, it may be difficult to generalize the results of this review to LMIC settings where the legal and healthcare payment contexts are drastically different. Travel for abortion in developing countries, compounded with other barriers to care, could have a disproportionate impact on women’s health compared to countries where abortion is legal; additional studies should explore this as a dimension of access. As access to abortion (and health care in general), should be within reach for all individuals, regardless of context, adaptations of recommended measures, such as travel time, could highlight disparities and barriers to abortion access that women all over the world face.

### Limitations

There are some potential limitations of this systematic review. The majority of studies in the review are US-based which may make the findings difficult to generalize, particularly to settings where abortion is not legal. It may also be difficult to standardize and compare travel distance and costs globally; more research from non-US contexts is needed to address this issue. The studies in this review represent a range of designs, including modeling studies, those that contain national and state data, and studies involving discrete groups of women who travel for abortion. For this reason, comparisons across studies may be difficult, but because of the lack of available literature, all types of studies around travel for abortion were included to construct a complete summary of the research on this topic. Finally, the populations in the reviewed studies were women who traveled for abortions; there are no studies about women who did not make it to clinics or who chose to self-induce. Recent research shows that this may make up a considerable portion of women seeking abortions [81–82]; the experiences of these women around travel for abortion would contribute substantially to our understanding of barriers to abortion care.

## Conclusion

This systematic review synthesizes the literature on the experiences of women who travel for abortion, including 46 quantitative studies, 12 qualitative studies, and one mixed methods study. Travel was categorized as a primary focus for 15 studies, a secondary focus for 27 studies, and a tertiary focus for 17 studies. Quality of studies was generally high, with participant flow and potential biases identified as areas for improvement. Travel distance, cost, and time were identified as burdens of travel for abortion care. Future studies should consider the use of standardized travel distance and cost measurements and continue to explore additional dimensions of the burdens of abortion related travel on the lives of women who need abortions.

## Appendices

**Appendix 1**. Search strategy details. (Please see file Appendix 1 at [DOI:10.7272/Q6JW8C2G]).

**Appendix 2.** Quantitative data extraction. (Please see file Appendix 2 at [DOI:10.7272/Q6JW8C2G]).

**Appendix 3.** Qualitative data extraction. (Please see file Appendix 3 at [DOI:10.7272/Q6JW8C2G]).

**Appendix 4.** STROBE checklist data (randomized). (Please see file Appendix 4 at [DOI:10.7272/Q6JW8C2G]).

**Appendix 5.** CASP checklist data (randomized). (Please see file Appendix 5 at [DOI:10.7272/Q6JW8C2G]).

**Appendix 6.** Qualitative analysis codebooks. (Please see file Appendix 6 at [DOI:10.7272/Q6JW8C2G]).

## Supporting information

S1 PRISMA Checklist (Please see file S1 at [DOI:10.7272/Q6JW8C2G]). (DOC)
